# Supplementary material for: Antibiotic Prescribing Practices in Endodontic Infections: A Survey of Dentists in Serbia
Source: Antibiotics (Basel). 2021 Jan 12;10(1):67. doi: 10.3390/antibiotics10010067 (PMC7827796; doi:10.3390/antibiotics10010067)
Supplement: Supplementary file 1 [file antibiotics-10-00067-s001.pdf]

File S1. Questionnaire

Antibiotic prescription in endodontic infections - questionnaire

1. Gender: □ Male □ Female
2. Age (yrs): □ 25−35 □ 36−45 □ 46−55 □ 56−65
3. Academic degree: □ doctor of dentistry □ master of endodontics □ master in other branch of dentistry
4. Which antibiotic do you prescribe most often for an adult patient with no medical allergies?
   - Amoxicillin (Amoksicilin®, Ospamox®, Sinacillin®): □ 250 mg □ 500 mg □ 1000 mg
   -Amoxicillin+Clavulanic acid (Amoksiklav®, Augmentin®, Betaklav®, Klavobel®, Panklav®): □ 500+125 mg □ 875+125 mg □ 1000+62.5 mg
   -Clindamycin (Clindamycin MIP®): □ 300 mg □ 600 mg
   -Azithromycin (Azitromicin®, Sumamed®, Hemomycin®, Azibiot®): □ 250 mg □ 500 mg
   -Metronidazole (Orvagyl®): □ 250 mg □ 400 mg
   -Other:__________________
5. When you prescribe antibiotics, how long is the duration of treatment:
   □ 3 days
   □ 5 days
   □ 7 days
   □ 10 days
   □ until symptoms disappear
6. Which antibiotic do you prescribe most often for an adult patient with no allergies to penicillin?
   □ Clindamycin (Clindamycin MIP®)
   □ Azithromycin (Azitromicin®, Sumamed®, Hemomycin®, Azibiot®)
   □ Metronidazole (Orvagyl®)
   □ Erithromycin (Eritromicin HF®)
   □ Tetracycline (Amracin®)
   □ Other:__________________
7. In which situations do you prescribe antibiotics (more than one answer is allowed):
   □ irreversible pulpitis (pain, no other symptoms of infection)
   □ pulp necrosis (non-vital teeth, widening of periodontal space)
   □ acute apical periodontitis (pain, pain to percussion, widening of periodontal space)
   □ chronic apical abscess (widening of periodontal space, teeth with sinus tract)
   □ acute apical abscess with no systemic involvement (localized fluctuant swellings)
   □ acute apical abscess in medically compromised patients (localized fluctuant swellings, patient with systemic disease causing impaired immunologic function)
   □ acute apical abscess with systemic involvement (localized fluctuant swellings, elevated body temperature >38 °C, malaise, lymphadenopathy, trismus)
   □ progressive infections (rapid onset of severe infection/within 24 h, cellulitis)
   □ persistent infections (osteomyelitis, chronic exudation)
   □ post-operative pain
   □ always during endodontic treatment
   □ perforation

Thank you for your collaboration!
